# Pyrolysis of Softwood Carbohydrates in a Fluidized Bed Reactor

**DOI:** 10.3390/ijms9091665

**Published:** 2008-09-02

**Authors:** Atte Aho, Narendra Kumar, Kari Eränen, Bjarne Holmbom, Mikko Hupa, Tapio Salmi, Dmitry Yu. Murzin

**Affiliations:** 1 Laboratory of Industrial Chemistry and Reaction Engineering, Process Chemistry Centre, Åbo Akademi University, FI-20500 Åbo/Turku, Finland; 2 Laboratory of Wood and Paper Chemistry, Process Chemistry Centre, Åbo Akademi University, FI-20500 Åbo/Turku, Finland; 3 Laboratory of Inorganic Chemistry, Process Chemistry Centre, Åbo Akademi University, FI-20500 Åbo/Turku, Finland

**Keywords:** Pyrolysis, cellulose, galactoglucomannan, pine, fluidized bed reactor, bio-oil

## Abstract

In the present work pyrolysis of pure pine wood and softwood carbohydrates, namely cellulose and galactoglucomannan (the major hemicellulose in coniferous wood), was conducted in a batch mode operated fluidized bed reactor. Temperature ramping (5 °C/min) was applied to the heating until a reactor temperature of 460 °C was reached. Thereafter the temperature was kept until the release of non-condensable gases stopped. The different raw materials gave significantly different bio-oils. Levoglucosan was the dominant product in the cellulose pyrolysis oil. Acetic acid was found in the highest concentrations in both the galactoglucomannan and in the pine wood pyrolysis oils. Acetic acid is most likely formed by removal of *O*-acetyl groups from mannose units present in GGM structure.

## 1. Introduction

Liquid biofuels can be produced from woody biomass through pyrolysis, e.g. thermal degradation, which also gives, besides the liquid pyrolysis oil, non-condensable gases and a solid residue char. The process can be tailored for high production of one of the product phases. For instance, fast pyrolysis affords high liquid yields. Characteristics of fast pyrolysis, which could be conducted in a suitable fashion in a fluidized bed reactor, are high heating rates and short vapour residence times.

Several researchers [1–4, 7–13] have studied the pyrolysis of cellulose. Pyrolysis of cellulose is believed to follow the Broido-Shafizadeh model. The first step in this model is the formation of active cellulose (AC), followed by two competing reaction pathways: one producing char and gases and the other producing volatiles, mainly levoglucosan, through a transglycosylation reaction [1]. Levoglucosan can further be dehydrated forming levoglucosenone and 1,4:3,6-dianhydro-α-D-gluco-pyranose (DPG). Fission and disproportionation reactions can furthermore transform levoglucosan into furans and low-molecular-mass acids. Luo *et al.* [2] proposed a modified Broido-Shafizadeh (B-S) model. They suggested that degradation of AC would proceed along three competing reaction pathways. AC was thought to degrade into levoglucosan and to char and gases, as in the B-S model. A new competing pathway was suggested for the formation of hydroxy-acetaldehyde and 1-hydroxy-2-propanone from AC.

Yang *et al.* [3] studied the pyrolysis of the three major biomass components, namely, cellulose, hemicellulose and lignin by thermogravimetric analysis. They used xylan as a model component for hemicellulose. Pyrolysis of xylan started at 220 °C and reached a maximum weight loss rate at 260 °C. The thermal degradation of cellulose occurred at slightly higher temperature. Pyrolysis of cellulose started at 315 °C and had a maximum weight loss rate at 355 °C.

Woody biomass is known to contain high amounts of ash-forming matter. These alkali metals have been shown to influence the choice of cellulose pyrolysis reaction pathway [4–6]. Essig *et al.* [4] noticed that small amount of added salt to cellulose suppressed the levoglucosan forming reaction pathway, instead the formation of hydroxyacetaldehyde and 1-hydroxy-2-propanone increased.

The purpose of this work was to study the slow pyrolysis of the main softwood carbohydrates, namely, cellulose and galactoglucomannan. It should be noted that there is no data reported in the literature for pyrolysis of galactoglucomannan.

## 2. Experimental Section

### 2.1. Materials

Galactoglucomannan (GGM) is the major hemicellulose in softwood. The backbone of GGM is believed to be straight and is built up of glucose and mannose units. Single-unit side chains of galactose are attached to the backbone. C-2 and C-3 of the mannose are partly substituted by *O*-acetyl groups. Due to these substitutions GGM is water soluble [14]. The GGM used in this work was provided by the Laboratory of Wood and Paper Chemistry at Åbo Akademi and had been isolated from process water in a mill producing spruce thermomechanical pulp. Microcrystalline cellulose powder (Aldrich) and fresh pine wood from an about 20-year-old tree (*Pinus silvestris*) were also processed in the pyrolysis experiments.

### 2.2. Pyrolysis reactor

**Figure 1.**
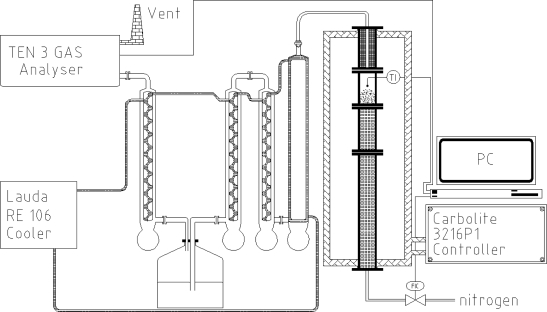
Pyrolysis reactor set-up.

Pyrolysis of softwood carbohydrates was conducted in a batch-operating fluidized bed reactor, illustrated in Figure 1. The inner diameter of the reactor was 34 mm and the height 79 mm. In principle the reactor set-up has a feeder attached to the reactor, however, during these experiments no feeder was used because of the sticky behavior of the GGM.

Quartz sand was used as bed material in the fluidized bed reactor. The particle size distribution of the sand was 100 – 150 μm. All sand was kept in the reactor by a net at the upper part of the reactor with a hole size of 45 μm. In addition it should be mentioned that no char was found in the bio oil.

The heating of the pyrolysis reactor was performed in a CARBOLITE split tube furnace model VST 12/400 and controlled by a 3216P1 model controller. Cooling of the evolved vapours was carried out in four consecutive coolers. Fluidization of the bed was achieved with 1 L/min of nitrogen, controlled by a Brooks^®^ 5850S mass flow controller. Between the third and fourth cooler the vapours were passed through a water quench, and the pH of the water was adjusted to 3 with nitric acid to avoid absorption of CO_2_. Thereafter, the uncondensed gases were analysed for CO, CO_2_ and hydrocarbons by a TEN_3_GAS analyser. The temperature inside the reactor was monitored by a K-type thermocouple. All temperature and gas data were logged to a computer.

**Figure 2.**
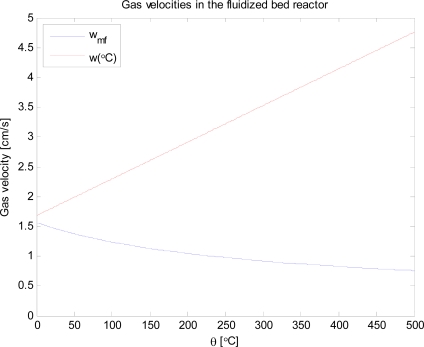
Minimum fluidization velocity and actual gas velocity in the reactor.

Fluidization of a fixed bed occurs when the pressure drop over the bed corresponds to the bed gravity. The minimum fluidization velocity can be calculated with equation derived by Ergun [15], which requires the values of the porosity of the fixed bed and the form factor. The latter is defined as the surface area of the equivalent sphere divided by the surface area of the actual particle that the bed is made up of. The values for the bed porosity and form factor used in the calculations are 0.499 and 0.632, respectively. The particle size used in the calculations was 125 μm and the density was 2653.5 kg m^−3^. By plotting the minimum fluidization velocity at different temperatures (taking into account dependence of the gas density and viscosity on temperature) and the velocity of the fluidization gas in Figure 2 it can be observed that fluidization is achieved at all applied temperatures.

The actual flow of nitrogen at 450 °C corresponds to 5.6 times the minimum fluidization velocity at this temperature.

### 2.2. Pyrolysis experiments

Pyrolysis of pine wood and softwood carbohydrates namely, cellulose and galactoglucomannan, was conducted in batch mode in the reactor set-up described above. The reactor was loaded with approximately 10 g of raw material and 30 g of quartz sand.

**Figure 3.**
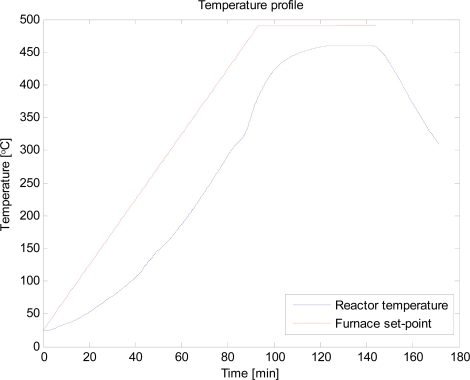
Temperature profile of the pyrolysis experiment.

The set point of the LAUDA cooling system was set to −20 °C. When the set point was reached the heating of the furnace started. The temperature ramp applied on the heating was 5 °C/min until 490 °C were reached. Thereafter the temperature was kept at 490 °C until the release of non-condensable gases stopped. When the temperature of the furnace was 490 °C the temperature in the reactor was approximately 460 °C. These temperature conditions were found to be optimal in maximizing bio-oil yield in the previous work on catalytic pyrolysis of woody biomass [16, 17]. The temperature profile is shown in Figure 3. Nitrogen (1 L/min) was continuously fed through the bottom of the reactor for the fluidization of the bed and for purging of the evolved vapours. When no CO or CO_2_ was detected by the TEN_3_GAS analyser the heating of the furnace was discontinued.

After the experiment the amount of char was determined gravimetrically and the amount of non-condensable gases was calculated. The water content of the produced oil was determined by Karl Fischer titration and the chemical composition was analysed by GC-MS. The column used in the GC-MS was an Agilent DB-Petro 50 m column, with an inner diameter of 0.2 mm and a film thickness of 0.5 mm. The temperature program started at 40 °C and was kept isothermal for 10 min. Thereafter the temperature was raised to 75 °C with a ramp of 0.90 °C/min. When the temperature of the column was 75 °C, a steeper 1.10 °C/min ramp was used until reaching 120 °C. Finally the temperature was raised to 200 °C with a very steep 10 °C/min ramp. When the final temperature of 200 °C was reached it was kept isothermal for 20 min. The columns inlet pressure was 135 kPa and the scanning range was 10–300 amu. The bio oil was diluted in methanol in a way that the amount of oil was 0.2 g and the total weight of the diluted sample 0.5 g. The mixture was directly injected (1 μl) into the GC.

## 3. Results and Discussion

### 3.1. Pyrolysis results

Temperature programmed pyrolysis of softwood carbohydrates and pine wood produced three different product phases. The phases were classed as bio-oil, which contains condensed organic species and water, char and gases. The water content in the bio-oil (which appeared as one continuous phase) was determined by Karl Fischer titration and thereby the bio-oil could be split into oil and water. The raw material residue in the reactor was classed as char. CO and CO_2_ were analysed by the TEN_3_GAS analyser. The yield of the product phases, in wt-% calculated from the initial amount of raw material, are given in Table 1.

**Figure 4.**
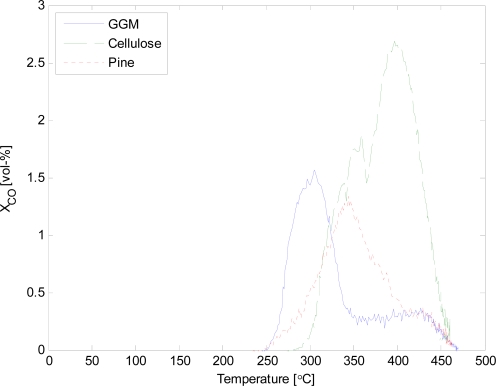
Concentration of CO as a function of temperature.

**Figure 5.**
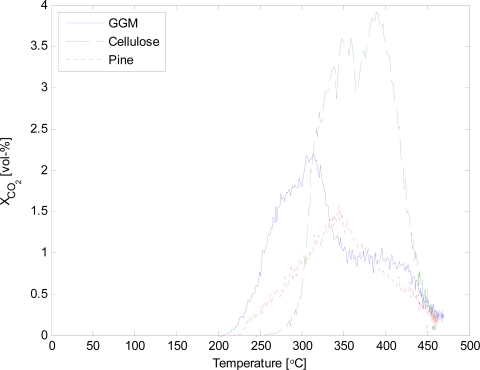
Concentration of CO_2_ as a function of temperature.

Unfortunately, the mass balance could not be closed with the current reactor set-up. The values for the gases and char can be considered reliable. However, the condensation of the vapours was insufficient. Continuous gas analysis made it possible to determine the release of different gases during pyrolysis at different temperatures. The volumetric concentration of CO and CO_2_ as a function of pyrolysis temperature are given in Figures 4 and 5.

The release of non-condensable gases from galactoglucomannan started at lower temperatures than for cellulose. The difference in temperature was approximately 50 °C for both CO and CO_2_. Pine wood consists of both cellulose and hemicellulose, especially GGM, as well as lignin. Thus, the thermal behaviour of pine is closely coupled to the individual components. The maximum release of CO, for pine wood, is between the maximum for GGM and cellulose. Similar observations can be made for the release of CO_2_. The total release of non-condensable gases, given in Table 1, was similar for both GGM and cellulose, while, for pine wood the amount of gases was lower. This could be due to the fact that about 30 wt-% of the pine wood is lignin.

**Figure 6.**
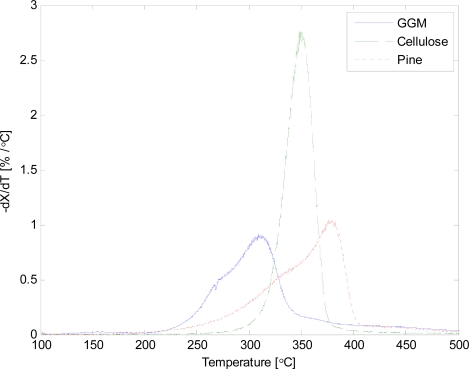
Normalized weight loss rate curves.

Thermogravimetric analysis (TGA) was performed on the different raw materials. The applied heating rate in the TGA was 20 °C/min, since higher heating rates change the temperature of the maximum weight loss rate. The normalized weight loss rate was calculated with equation (1). The result is illustrated in Figure 6.

(1) $$\frac{dX_{norm}}{dT}=\frac{X_{norm}(i+1)-X_{norm}(i)}{T(i+1)-T(i)}$$

The thermal degradation of the different materials occurred at different temperatures. The result is in accordance with Yang *et al.* [3]. The char yield corresponds well to the slow pyrolysis experiment for GGM and pine wood, contrary to cellulose which produced only 4.2% of char in TGA.

### 3.2. Chemical composition of the bio-oil

The composition of the bio-oil was analysed by GC-MS analysis. The gas chromatography area percent of the ten most dominating compounds in the different bio-oils is given in Tables 2 – 4.

The products found in the cellulose bio-oil correspond well to the literature [1]. Levoglucosan is probably formed from the active cellulose, or directly through transglycosylation. Further dehydration of levoglucosan produces 1,4:3,6-dianhydro-α-D-glucopyranose (DPG). Dehydration and decarbonylation of levoglucosan and DPG forms 2-furanmethanol. Furfural is likely formed through dehydration of 2-furanmethanol. Numerous dehydration reactions correspond well to the high water yield given in Table 1.

The mannose in GGM is partly substituted by *O*-acetyl groups. Acetic acid was the major chemical product formed in pyrolysis of GGM, since removal of the *O*-acetyl groups leads to acetic acid. The formation of levoglucosan from GGM is lower than from cellulose. This could be due to the fact that the GGM polymer is branched and amorphous, contrary to cellulose, and thereby inhibits the formation of levoglucosan through transglycosylation.

The high concentration of 1-hydroxy-2-propanone found in the pine wood pyrolysis oil could be explained by the presence of the alternative alkali-metal-catalyzed reaction pathway. The high number of phenolic compounds originates from the lignin polymer. In the case of wood pyrolysis, bio-oil could contain heavier pyrolytic lignin compounds. The presence of pyrolytic lignin has been investigated through the following procedure. When the bio oil was extracted with water most of the bio oil was soluble (90.1%) and the remaining 9.9% of the bio oil was analyzed by SEC confirming the small size of the molecules in the water insoluble part of the oil.

## 4. Conclusions

Slow pyrolysis of the main softwood carbohydrates, cellulose and galactoglucomannan (GGM), was conducted in a fluidized bed reactor. The different raw materials gave significantly different bio-oil. Pyrolysis of cellulose produced mainly levoglucosan, 1,4:3,6-dianhydro-α-D-glucopyranose (DGP) and 2-furanmethanol. The GC area percent of these compounds was over 50%. The higher ratio of CO to CO_2_ of cellulose compared to GGM was probably a result of decarbonylation of levoglucosan and DGP. Pyrolysis of GGM resulted mainly in acetic acid, 2-furanmethanol and levoglucosan. Acetic acid is most likely formed by removal of *O*-acetyl groups from mannose units present in GGM structure. The area percent of these compounds was only 17%. This indicates that GGM is degraded through several reaction pathways.

The composition of the pine wood pyrolysis oil reflects the three major chemical components of wood, namely cellulose, hemicellulose and lignin. The high concentration of 1-hydroxy-2-propanone is probably associated with the alternative alkali-metal-catalyzed reaction pathway.

## Figures and Tables

**Table 1.** Yield [wt-%] of pyrolysis product phases.

	Oil	Water	Char	CO	CO_2_	Total
GGM	12.1	18.6	31.2	2.3	12.5	76.7
Cellulose	23.1	24.5	20.1	4.7	10.5	82.9
Pine	25.1	14.8	21.5	2.5	6.7	70.6

**Table 2.** Chemical composition of cellulose bio-oil.

Area%	Library/ID
29.6	1,6-anhydro-β-D-glucopyranose (levoglucosan)
13.5	1,4:3,6-dianhydro-α-D-glucopyranose
12.0	2-furanmethanol
2.2	Furfural
2.1	1,6-anhydro-β-D-glucofuranose
1.7	5-(hydroxymethyl)-2-furancarboxaldehyde
1.4	(1R)-1-hydroxy-3,6-dioxabicyclo[3.2.1]octan-2-one
1.1	acetic acid
1.0	2-hydroxy-3-methyl-2-cyclopenten-1-one
0.8	5-methyl-furfural

**Table 3.** Chemical composition of GGM bio-oil.

Area%	Library/ID
6.2	acetic acid
6.1	2-furanmethanol
5.3	1,6-anhydro-β-D-glucopyranose (levoglucosan)
2.0	1-hydroxy-2-propanone
2.0	1,2-benzenediol
1.7	Furfural
1.7	1-(acetyloxy)-2-propanone
1.6	2-hydroxy-2-cyclopenten-1-one
1.5	dihydro-2(3H)-furanone
1.2	2-cyclopenten-1-one

**Table 4.** Chemical composition of pine wood bio-oil.

Area%	Library/ID
6.7	acetic acid
5.2	1-hydroxy-2-propanone
3.5	2-methoxy-4-propyl-phenol
3.4	1,6-anhydro-β-D-glucopyranose (levoglucosan)
2.7	2,5-dimethoxytetrahydrofuran
2.6	2-hydroxy-2-cyclopenten-1-one
2.1	2-methoxy-4-methyl-phenol
2.1	2-methoxy-4-(1-propenyl)-phenol
2.1	acrolein,dimethyl acetal
2.1	methyl formate
